# Hepcidin expression in human airway epithelial cells is regulated by interferon-γ

**DOI:** 10.1186/1465-9921-12-100

**Published:** 2011-08-02

**Authors:** Marie D Frazier, Lisa B Mamo, Andrew J Ghio, Jennifer L Turi

**Affiliations:** 1 Department of Pediatrics, Duke University Medical Center, Durham, NC 27710, USA; 2 Department of Pediatrics, Marshall University, Huntington, WV 25701, USA; 3 National Health and Environmental Effects Research Laboratory, Office of Research and Development, Environmental Protection Agency, Research Triangle Park, NC 27711, USA

## Abstract

**Background:**

Hepcidin serves as a major regulator of systemic iron metabolism and immune function. Airway epithelial cells have an extensive interface with the environment, and so must be able to respond locally to the presence of particulates, infection, and inflammation. Therefore, we hypothesized that hepcidin is expressed in airway epithelial cells and is regulated by early phase cytokines.

**Methods:**

Primary, differentiated human bronchial epithelial (NHBE) cells were used to assess hepcidin gene expression in response to IFN-γ, TNF-α, IL-1β, and IL-6, as well as to LPS + CD14. The role of the Janus Kinase-signal transducer and activator of transcription (JAK-STAT) pathway in IFN-γ-mediated hepcidin production was assessed by measuring JAK2 phophorylation and STAT1 nuclear translocation. Inductively coupled plasma mass spectroscopy (ICP-MS) was used to determine whether hepcidin altered iron transport in either NHBE cells or primary alveolar macrophages.

**Results:**

We demonstrate that differentiated human airway epithelial cells express hepcidin mRNA and that its expression is augmented in response to IFN-γ via activation of STAT1. However, while IFN-γ induced hepcidin gene expression, we were not able to demonstrate diminished expression of the iron export protein, ferroportin (Fpn), at the cell surface, or iron accumulation in airway epithelial in the presence of exogenous hepcidin.

**Conclusion:**

These data demonstrate that airway epithelial cells express hepcidin in the lung in response to IFN-γ. The presence of hepcidin in the airway does not appear to alter cellular iron transport, but may serve as a protective factor via its direct antimicrobial effects.

## Background

Hepcidin is a key regulator of systemic and cellular iron homeostasis. The 25-amino acid peptide is secreted predominantly by the liver in response to anemia, hypoxia, and inflammation [1-4]. Systemically, hepcidin coordinates iron absorption and export by binding to the iron export protein, ferroportin (Fpn), which is then phosphorylated, internalized, and ubiquitinated. The subsequent degradation of Fpn in the lysosomes leads to decreased cellular iron export and intracellular iron retention [5-8]. Locally, hepcidin is expressed in numerous cell types including macrophages, myocytes, and neurons, where it responds in a tissue specific manner to alterations in iron content, changes in oxygen tension, and the presence of inflammation [1,7,9-11].

In addition to its primary role in iron metabolism, hepcidin also plays an important role in immune function. Iron is an essential nutrient for growth and development of all organisms, and its availability closely correlates with bacterial virulence [12-14]. Hepcidin modulates immune function in part by its ability to decrease iron absorption and serum iron content in response to infection and inflammation in order to reduce the iron available to pathogens. Hepcidin also protects against infection via its ability to attack pathogens directly [15]. Initial identification of hepcidin, or liver-expressed antimicrobial peptide (LEAP-1), was based on the presence of structural features and a spectrum of antimicrobial activity that are very similar to the defensin peptide family [3,15]. Antimicrobial peptides serve as an important component of the innate immune system and are predominantly expressed at epithelial surfaces where interactions with the outside environment exist and therefore constitute the first line of defense against invading pathogens [16].

The lungs provide an extensive interface with the environment, and therefore, are continually exposed to inhaled iron-containing particulates and airborne microbials. As the first line of defense, airway epithelial cells, together with macrophages, must provide a coordinated system of defense. An essential component of this is to prevent the unregulated access of host metal to microbes [17]. The pattern of regulation of the iron export protein, Fpn, in the lung suggests that it serves an iron detoxification function rather than the nutritive purpose of iron transporters in the duodenal epithelial cells [18]. Given the lungs' extensive interface with the environment and its subsequent need for local control of iron accessibility and immune function, we hypothesized that hepcidin is expressed by airway epithelial cells. We further postulated that hepcidin expression is coordinated by pro-inflammatory cytokines to provide a localized mechanism to augment the antimicrobial defense of the airway.

## Methods

### Cell culture

Primary human bronchial epithelial (NHBE) cells from three normal donors were obtained from Lonza (Walkersville, MD) and expanded to passage-3 in bronchial epithelial cell basal medium (BEBM). Cells were plated on collagen-coated (rat-tail collagen, type I, 50 ug/ml/0.02 N acetic acid) Transwell^® ^filter supports (24.5 mm, 0.45 μm; Corning Costar, Cambridge, MA) at a density of 1 × 10^5 ^cells/filter in a 6-well culture plate format and maintained in BEGM medium as a 1:1 mixture of BEBM and Dulbecco's Modified Eagle Medium with high glucose (DMEM-H) containing hydrocortisone (0.5 μg/ml), epinephrine (0.5 μg/ml), insulin (5 μg/ml), triiodothyronine (6.5 ng/ml), transferrin (10 μg/ml), gentamicin (50 mg/ml), amphotericin-B (50 ng/ml), bovine pituitary extract (13 mg/ml), bovine serum albumin (1.5 μg/ml), nystatin (10,000 U), hEGF (25 ng/ml), and retinoic acid (5 × 10^-5 ^M) as previously described [19]. Fresh medium was provided every 48 hours. Upon reaching 75% confluence, the apical medium was removed and the cells maintained at air-liquid interface (ALI) until they achieved uniform differentiation into mucociliary epithelium after approximately 14 days post ALI.

Alveolar macrophages were acquired from healthy, nonsmoking volunteers (18-40 years of age) by fiber-optic bronchoscopy with bronchoalveolar lavage. The protocol and consent form were approved by the University of North Carolina School of Medicine Committee on the Protection of the Rights of Human Subjects. The fiber-optic bronchoscope was wedged into a segmental bronchus of the lingula and then the right middle lobe. Aliquots of sterile saline were instilled, immediately aspirated, and centrifuged. Macrophages from aliquots were pooled and washed twice. Incubations were in RPMI-1640 (Invitrogen, Carlsbad, CA, USA) supplemented with 10% fetal calf serum (FCS; Invitrogen) and gentamicin solution (20 μg/ml; Sigma, St. Louis, MO, USA).

### In vitro exposure

Differentiated NHBE cells were exposed apically to Hank's balanced salt solution (HBSS) as control; lipopolysaccharide (LPS, Escherchia coli 055:B5/L 4005; 100 μg/ml; Sigma, St. Louis, MO) and CD14 (250 ng/ml; Cell Sciences, Canton, MA); cytomix (IL-1β, 100 ng/ml, Fitzgerald Industries, Concord, MA; TNF-α, 100 ng/ml; IFN-γ, 100 ng/ml, R&D Systems, Minneapolis, MN); or IL-6 (100 ng/ml; R&D Systems). These doses have previously been demonstrated to significantly alter regulation of iron transport [20]. Absence of toxicity with this dosing was verified using measurement of LDH release. The cells were exposed for 1 hour, the reagent removed, and the cells rinsed with HBSS. Cells were harvested for measurement of RNA expression 2, 6, or 24 hours after exposure or for JAK2 activation or STAT1 phosphorylation 15, 60, or 120 minutes after exposure.

Additional cells were pretreated with an inhibitor of JAK2 phosphorylation, AG490 (10 μM; Calbiochem, San Diego, CA) for 1 hour followed by treatment with IFN-γ for 1 hour. Cells were harvested for measurement of RNA expression 24 hours after exposure. The role of IL-6 was measured by pre-treating differentiated NHBE cells with a soluble IL-6 receptor (IL-6sR, 1 μ/ml; R&D Systems) for one hour prior to treatment with either IFN-γ or IL-6 for an additional hour. Cells were harvested for measurement of RNA expression 2 or 24 hours after exposure.

### RNA isolation and RT-PCR analysis

Total RNA was isolated from NHBE cells by RNeasy^® ^(Qiagen, Valencia, CA). Briefly, cells were rinsed with HBSS and immediately lysed and homogenized in guanidine isothiocyanate (GITC)-containing buffer, precipitated by ethanol, bound on a column, washed, and eluted with water. Following reverse transcription of total RNA, the resulting cDNA was amplified by Real Time PCR (ABI Prism 7700 Sequence Detector System, Applied Biosystems, Foster City, CA). Primers were chosen to span introns to minimize genomic amplification. The hepcidin primer pair and fluorescent probes were designed and produced by Applied Biosystems (accession no. NM_021175). 18S primer pair and fluorescent probe (TaqMan^® ^Ribosomal RNA Control Reagents) were obtained from Applied Biosystems. IL-6 primer pair and fluorescent probe were designed using the following sequences: Forward: 5' GGT ACA TCC TCG ACG GCA TCT 3'; Reverse: 5' GTG CCT CTT TGC TGC TTT CAC 3'; Probe: 5' TGT TAC TCT TGT TAC ATG TCT CCT TTC TCA GGG CT 3'. Relative quantification of hepcidin gene expression by Real Time PCR was performed by fluorogenic amplification of cDNA using a TaqMan Universal PCR Master Mix (Applied Biosystems). Relative quantification of hepcidin, IL-6, and 18s was based on standard curves prepared from the serially diluted cDNA of primary macrophages for hepcidin and BEAS2B cells for IL-6 and 18s. The abundance of hepcidin and IL-6 mRNA in each sample was standardized against that of 18s rRNA.

### Isolation of total cellular protein

Cells were scraped and lysed with RIPA lysis buffer (1% Igepal, 0.5% deoxycholate, 0.1% SDS/PBS, pH 7.4) containing protease (Invitrogen,) and phosphatase inhibitors (Sigma). Cells were sheared through a 22-gauge needle and the cellular debris was pelleted by centrifugation at 14,000 × rpm for 5 minutes. The supernatant was removed and protein content determined using the Lowry DC Protein Assay (BioRad, Hercules, CA).

### Isolation of nuclear protein

NHBE cells were rinsed with ice cold 1x PBS, scraped into cytoplasmic extraction buffer (CEB; Tris-HCl 0.01 M, pH 7.9, KCl 0.06 M, EDTA 0.001 M, DTT 0.001 M,) with proteases and phosphatase inhibitors and incubated on ice for 15 minutes. Igepal was added to a final concentration of 0.1% and the samples were vortexed and spun at 14,000 × rpm for 1 minute at 4°C. The cell pellet was washed with CEB buffer and spun as above. The pellet was incubated in nuclear extraction buffer (NEB; Tris-HCL, pH 8.0 0.02 M, NaCl 0.4 M, MgCl_2 _0.0015 M, EDTA 0.0015 M, DTT 0.001 M, glycerol 25%) with proteases and phosphatase inhibitors for 10 minutes and spun at 14,000 × rpm for 5 minutes at 4°C. The nuclear fraction was assayed with Lowry DC reagent.

### Biotinylation

Two hours after treatment with hepcidin peptide or 24 hours after treatment with IFN-γ, NHBE cells were rinsed three times with ice cold 1x PBS, pH 8.0. EZ-Link^®^. Sulfo-NHS-LC-Biotin (500 ul, 2 mM in 1x cold PBS; Pierce Biotechnology, Rockford, IL) was added to each well and incubated at room temperature for 30 minutes. After incubation, the Biotin link was aspirated and the cells were washed three times with cold 10 mM glycine/1xPBS. Lysis buffer (0.05% Triton-X, 1 mM vanadium sulfate oxide, 1x PBS, pH 7.4 with anti-proteases) was added to each well and the cells removed by scraping. After gentle centrifugation to pellet debris, the cell supernatant was collected and concentration determined by Lowry DC protein assay (BioRad). The lysate concentration was standardized before SoftLink™ Soft Release Avidin Resin (100 ul/0.6 mg; Promega, Madison, WI) addition and incubated by end-over-end mixing for 3 hours at 4°C. Resin was allowed to settle and the lysate removed as above. The resin was then rinsed twice with sodium phosphate buffer (0.1M Na_2_HPO_4_, pH 7.2) with end-over-end mixing for 10 minutes. Biotinlyated surface proteins were eluted with 5 mM biotin (2x biotin/1x resin) by end-over-end mixing overnight at 4°C. Resin was allowed to settle and the protein removed with a 1 ml/27G syringe.

### Western blot analysis

Protein concentrations were equalized and loaded with 1X laemmli buffer (75 mM Tris-HCl, pH 6.8, 8% glycerol, 6% SDS, 9% β-mercaptoethanol, 0.05% bromophenol blue), separated by SDS-polyacrylamide gel electrophoresis, and transferred to a PVDF membrane. Membranes were blocked with 5% (wt/vol) blotting grade milk in TBST (0.05 M Tris, 0.138 M NaCl, 0.0027 M KCl pH 8.0, 0.05% Tween 20) for 1 hour at room temperature. Membranes were rinsed in TBST and incubated with the appropriate primary antibody ∝-JAK2, ∝-phos-JAK2, or ∝-phos-STAT1(Ser) (1:1000, 1:2000, or 1:1000, respectively; Cell Signaling, Danvers, MA); ∝-ferroportin (1:1000; generously provided by J. Kaplan, University of Utah), ∝-β-actin (1:20,000, Sigma, St.Louis MO) or ∝-tubulin (1:1000; Sigma) in 5% BSA or milk/TBST, overnight at 4°C. Antigen-antibody complexes were incubated with horseradish peroxidase - conjugated goat anti-rabbit IgG or goat anti-mouse IgG secondary (1:2000) for 1 hour in 5% milk/TBST at room temperature, rinsed, and detected using enhanced chemiluminescence (ECL, GE Healthcare, Piscataway, NJ). Both pre-stained and biotinylated markers were used to confirm transfer and molecular weight, respectively. Equal loading of protein was confirmed using Ponceau-S solution (Sigma). Expression was quantified using densitometry (GS-750, BioRad, Hercules, CA).

### Inductively coupled plasma mass spectroscopy (ICP-MS)

Cellular iron transport was measured by ICP-MS in differentiated NHBE cells at ALI and in alveolar macrophages. Cells were loaded with ^57^Fe by incubating the cells in 100 μM ^57^ferric ammonium citrate (FAC) for four hours. The cells were washed and media replaced. Cells were then treated with hepcidin peptide (1 μM; Peptide Institute, Osaka, Japan) for 20 hours. Media was removed and the cells washed and scraped into 3 N HCl/10% trichloroacetic acid. After hydrolysis at 70°C for 24 hours, ^57^Fe concentration in the acid lysate was quantified on a Perkin Elmer Elan 6000 inductively coupled plasma mass spectrometer. Indium was utilized as an internal standard.

### Statistical analysis

Statistical analysis was performed using Prism 4.0 (GraphPad Software, Inc., San Diego, CA). Expression data are displayed as fold change over control and iron transport data as the mean ratio of ^57^Fe/^56^Fe ± standard error (SE) (n = 4, unless otherwise specified). All experiments were performed at least in duplicate using two separate cell donors. Differences between multiple groups were compared using one-way analysis of variance (ANOVA) followed by Tukey's multiple comparison post hoc test. Two-way ANOVA was used to assess the effect of the response over time. Two-tailed tests of significance were employed. Significance was assumed at P < 0.05.

## Results

### Hepcidin is expressed in airway epithelial cells and is regulated by IFN-γ

**Figure 1:**
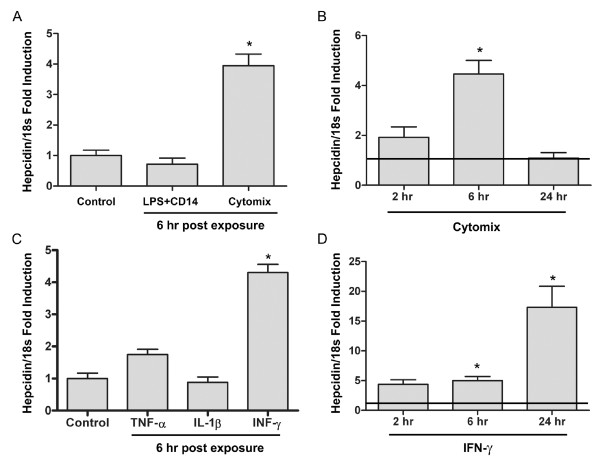
**Hepcidin gene expression is increased in airway epithelial cells after exposure to pro-inflammatory cytokines**. NHBE cells, grown at ALI, were exposed to LPS (100 μg/ml) + CD14 (250 ng/ml) or to cytomix (TNF-α, IL1-β, and IFN-γ (100 ng/ml, each)) for 1 hr and harvested after 6 hrs (A) or after 2, 6, and 24 hrs to establish a time response curve (B). Additional cells were exposed to individual cytokines, TNF-α, IL1-β, and IFN-γ for 1 hr and harvested after 6 hrs (C) or IFN-γ for 1 hr and harvested after 2, 6, and 24 hrs and compared to time-based controls (D). Total RNA was isolated using RNeasy^® ^and reversed transcribed. Quantitative PCR was performed using TaqMan polymerase. Fluorescence was detected on an ABI Prism 7700 sequence detector (Applied Biosystems). Relative abundance of hepcidin was normalized to 18s and expressed as fold induction over control ± SE and represent at least n = 4. * P < 0.001, ^#^P < 0.05 relative to time-based HBSS control cells.

To determine whether hepcidin is expressed locally in airway epithelial cells and is regulated in response to pro-inflammatory stimuli, we exposed differentiated NHBE cells to LPS (100 μg/ml) and CD14 (250 ng/ml) (the latter was employed to augment the response of the airway epithelial cells to LPS) [21], or to a mixture of pro-inflammatory cytokines (cytomix: TNF-α, IL-1β and IFN-γ (100 ng/ml each)). Changes in hepcidin mRNA levels were measured using Quantitative Real Time PCR. Constitutive expression of hepcidin mRNA was low at baseline but increased by six-fold in the presence of the pro-inflammatory cytokines (figure 1A) with a peak effect seen at 6 hours and a return to baseline 24 hours after exposure (figure 1B). No change in hepcidin mRNA expression was evident after exposure to LPS and CD14.

To better characterize the regulation of hepcidin by pro-inflammatory cytokines, NHBE cells were exposed individually to TNF-α, IL-1β, or IFN-γ. Hepcidin mRNA expression was found to increase after exposure to IFN-γ (figure 1C); with no significant effect by either TNF-α or IL-1β on hepcidin mRNA levels. The magnitude of change due to IFN-γ at 6 hours was essentially the same as that observed after cytomix. However, despite the return to baseline after exposure to cytomix, cells exposed to IFN-γ alone demonstrated a continued increase in hepcidin gene expression 24 hours after exposure (figure 1D).

### Regulation of hepcidin by IFN-γ is mediated via STAT1

**Figure 2:**
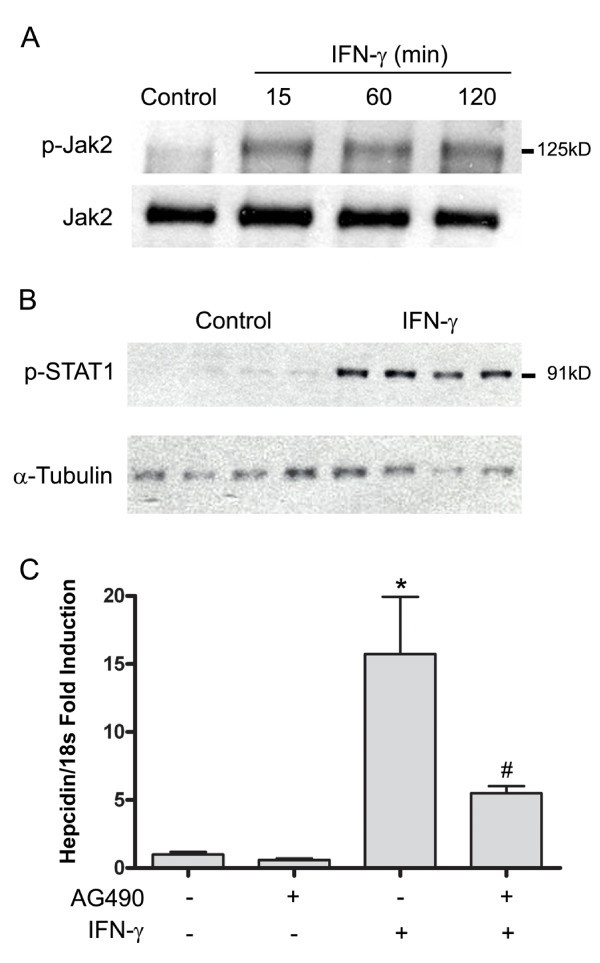
**IFN-γ activates the JAK-STAT pathway, which contributes to hepcidin induction**. NHBE cells, grown at ALI, were exposed to IFN-γ (100 ng/ml) for 15, 60, and 120 minutes. Whole cell protein lysates (40 μg) were separated by SDS-polyacrylamide gel electrophoresis (7.5%) followed by immunoblotting using a phos-JAK2 antibody (Cell Signaling, Danvers, MA; 1:2000) then stripped and re-probed using a JAK2 antibody (Cell Signaling, 1:1000) to assess total JAK2 expression (A). Nuclear protein extracts (5 μg) from additional NHBE cells exposed to IFN-γ for 15 minutes were probed with a phos-STAT1 antibody and standardized to α-tubulin (B). NHBE cells pre-treated with a JAK2 inhibitor (AG490, 10 μM) prior to exposure to IFN-γ were harvested 24 hrs following exposure. RNA was isolated, reversed transcribed, and quantitative PCR performed. Relative abundance of hepcidin was normalized to 18s and expressed as fold change over induction ± SE and represent n = 4. * P < 0.001 relative to HBSS control cells, ^#^P < 0.05 relative to IFN-γ stimulated cells.

Signaling via the IFN-γ receptor requires phosphorylation of the associated tyrosine kinases 1 and 2 which then mediate phosphorylation and activation of STAT1. Therefore, to determine the role of the JAK-STAT pathway in IFN-γ-induced hepcidin expression, we measured phosphorylated JAK2 in the presence and absence of IFN-γ in differentiated NHBE cells. Significant JAK2 phosphorylation occurred within 15 minutes of exposure to IFN-γ with continued activation through the 120 minute exposure (figure 2A). Moreover, increased phosphorylated STAT1 was evident in the nucleus of NHBE cells 15 minutes after exposure to IFN-γ (figure 2B). To more directly link the activation of the JAK-STAT1 pathway with regulation of hepcidin by IFN-γ, we pre-treated NHBE cells with AG490, an inhibitor of JAK2 phosphorylation. Inhibition of JAK2 phospohorylation significantly attenuated hepcidin induction in these cells 24 hours after exposure to IFN-γ (figure 2C).

### IFN-γ induces hepcidin expression independently of IL-6

**Figure 3:**
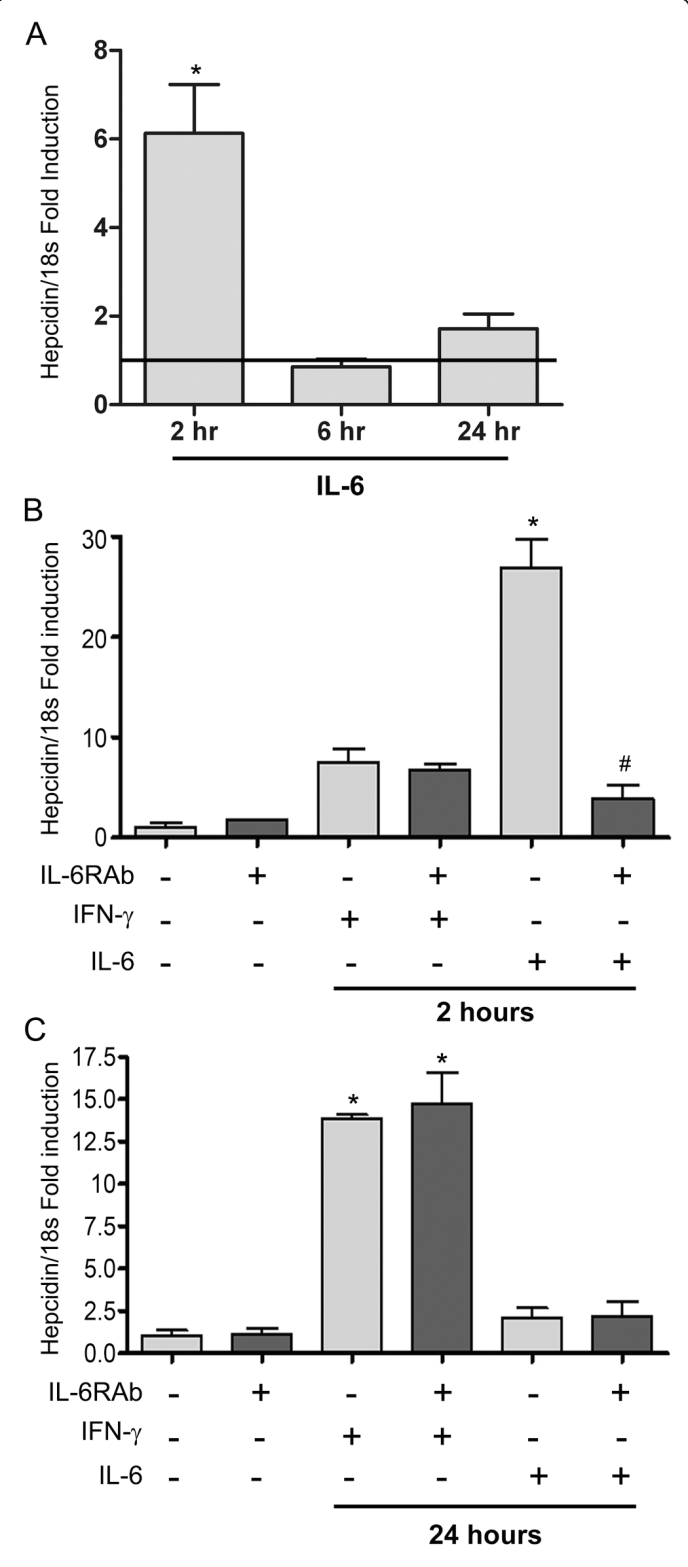
**IL-6 increases hepcidin gene expression in airway epithelial cells but does not contribute to IFN-γ induced hepcidin expression**. NHBE cells, grown at ALI, were exposed to IL-6 (100 ng/ml) for 1 hour and harvested after 2, 6, and 24 hrs (A). Additional NHBE cells were pre-treated with an IL-6 soluble receptor antibody (IL-6sR; 1 μg/ml) for 1 hr prior to exposure to either IFN-γ or IL-6, then harvested after 2 hrs (B) and 24 hrs (C). Hepcidin gene expression was measured by real time PCR, normalized to 18s, and expressed as fold induction over control ± SE (n = 4). * P < 0.001 relative to control; ^#^P < 0.05 relative to IL-6 stimulated cells.

IL-6 mediates systemic hepcidin expression and can be induced by IFN-γ [22,23]. To evaluate whether IL-6 plays a role in IFN-γ-mediated hepcidin expression, we first confirmed that IL-6 induces hepcidin gene expression in differentiated NHBE cells. We found that hepcidin mRNA expression rapidly increased with a peak effect 2 hours after stimulation with IL-6 (figure 3A) and return to baseline after 6 hours. To assess whether IFN-γ mediates hepcidin production by augmenting IL-6 expression, we pre-treated NHBE cells with an IL-6 receptor antibody prior to exposure to IFN-γ or IL-6 to block signaling through the IL-6 pathway. The mRNA levels measured 2 hours after exposure indicated that while the IL-6 receptor antibody abrogated IL-6-mediated hepcidin expression, it did not affect expression mediated by IFN-γ (figure 3B). Maximal induction of hepcidin by IFN-γ 24 hours after exposure likewise was not affected in the presence of the IL-6 receptor antibody (figure 3C).

### Hepcidin does not decrease Fpn expression at the cell surface

**Figure 4:**
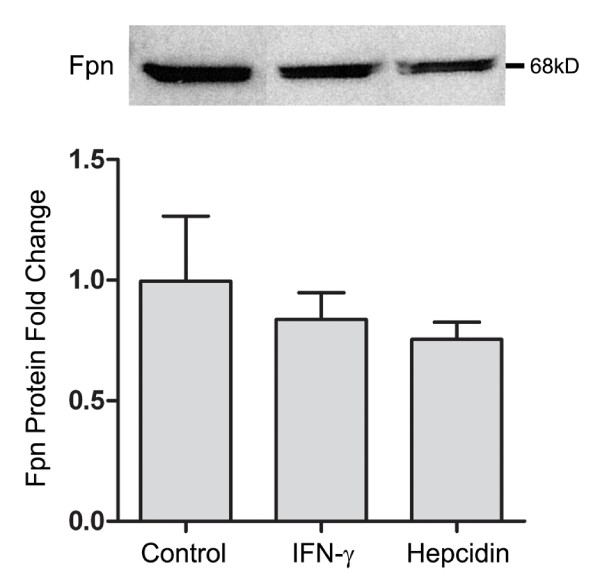
**Fpn surface expression is unchanged in the presence of exogenous hepcidin or IFN-γ**. NHBE cells, grown at ALI, were pre-treated with either IFN-γ for 24 hours prior or exogenous hepcidin peptide (1 μM) for 2 hours prior to biotinylation of cell surface proteins and cell harvest. Equal quantities of protein (300 μg) were separated by SDS-polyacrylamied gel electrophoresis (12%) followed by immunoblotting using an Fpn antibody (1:1000, generously provided by J. Kaplan, University of Utah) and standardized to Coumassie staining.

Systemically, the hepcidin protein acts to limit cellular iron export by internalizing and degrading the iron export protein, Fpn, which results in intracellular iron accumulation. We examined the effect of increased hepcidin on cell surface expression of Fpn in NHBE cells treated with either IFN-γ for 24 hours or exogenous hepcidin for 2 hours at a concentration consistent with measurements of serum hepcidin in patients with anemia of chronic disease or iron overload [24]. Using surface biotinylation and avidin precipitation, we found a small, though not statistically significant decrease in cell surface Fpn protein expression in the presence of either IFN-γ or hepcidin. This suggests that although hepcidin is present in NHBE cells and is regulated by inflammatory cytokines, it does not appear to significantly alter cell surface expression of the iron export protein within these experimental conditions (figure 4).

### Hepcidin does not alter iron transport in the airway

**Figure 5:**
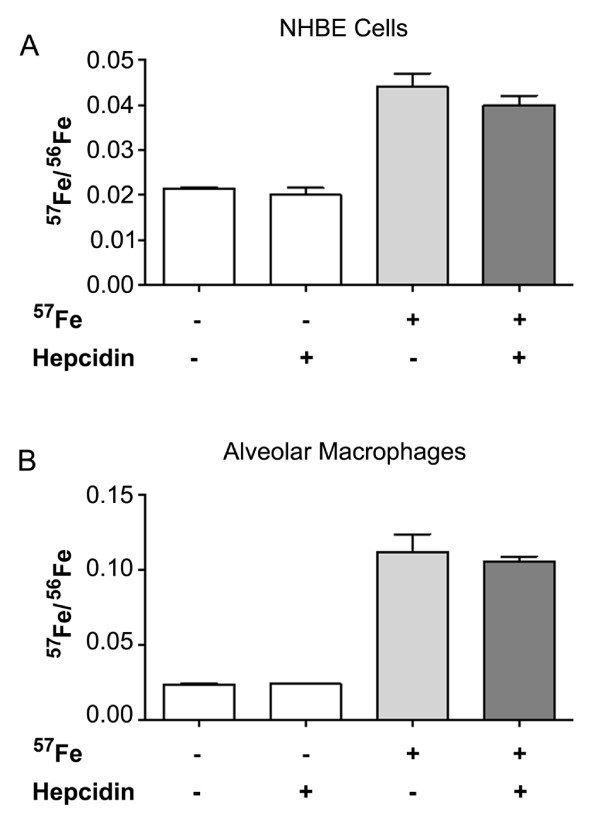
**Exogenous hepcidin does not alter iron transport in NHBE cells or alveolar macrophages**. Differentiated NHBE cells (A) or alveolar macrophages (B) were loaded with ^57^Fe for 4 hours, followed by treatment with exogenous hepcidin peptide (1 μM) for an additional 20 hours. Cells were washed and scraped into 3 N HCl/10% trichloroacetic acid. After hydrolysis at 70°C for 24 hours, ^57^Fe was quantified. The quantity of exogenous ^57^Fe was compared to the naturally occurring ^56^Fe and showed no change in either the airway epithelial cells or alveolar macrophages treated with exogenous hepcidin peptide.

We next evaluated the functional impact of increased hepcidin expression on cellular iron content. Differentiated NHBE cells were loaded with ^57^Fe for 4 hours, then treated with exogenous hepcidin for 20 hours. This duration of treatment was chosen because the uptake of iron by NHBE cells after exposure to Fe^3+ ^typically is accomplished over 4 hours with subsequent cellular export over the next 16 hours [18]. We found that, consistent with minimal internalization of Fpn with both IFN-γ and hepcidin, iron did not accumulate intracellularly in the presence of exogenous hepcidin (figure 5A). Because iron metabolism in the lung requires coordination between epithelial cells and macrophages [25], we next evaluated whether hepcidin released by airway epithelial cells could impact iron transport in macrophages. Freshly collected macrophages were obtained from healthy volunteers by bronchoscopy and loaded with ^57^Fe as described for NHBE cells. The macrophages were then incubated with hepcidin peptide for an additional 20 hours. No change in intracellular iron content was seen in macrophages treated with hepcidin (figure 5B).

## Discussion

Hepcidin is a major regulator of both iron metabolism and immune function. Given the constant exposure of the lungs to both metal-rich particles and microbes, we evaluated localized expression and regulation of hepcidin in the airway by inflammatory stimuli. We were able to demonstrate that hepcidin was expressed locally by airway epithelial cells, and that its expression can be regulated by the pro-inflammatory cytokine IFN-γ via activation of STAT1. However, while IFN-γ induced hepcidin gene expression, it was not associated with significantly decreased Fpn expression at the cell surface. Further, cellular iron accumulation in airway epithelial cells and alveolar macrophages was not altered by the presence of exogenous hepcidin using these experimental conditions.

The hepcidin peptide is predominantly synthesized in the liver where it is released into the circulation to control systemic iron metabolism in response to serum iron content and the presence of hypoxia or inflammation [1,26,27]. Locally, hepcidin is expressed by a number of cell types, including airway macrophages, monocytes, cardiac myocytes, and neurons where it may be differentially regulated, likely reflecting the need to locally control iron transport due to infection, ischemia, or iron accumulation [7,9-11,26]. Here, we found that hepcidin was expressed by airway epithelial cells and was induced by both IFN-γ and IL-6 in a cell-specific pattern. While response to IL-6 has been established in a number of cell types [10,26], hepcidin induction by IFN-γ alone has not been reported. Hepcidin expression in response to IFN-γ demonstrated more prolonged activation than the response to cytomix. This suggests that the presence of TNF-α or IL-1β may serve a modulatory role in hepcidin regulation. This pattern is consistent with that seen in blood monocytes and in HepG2 cells where TNF-α had minimal effect on basal expression of hepcidin, but significantly inhibited IL-6-mediated hepcidin induction [28]. Our cells also showed differential regulation of hepcidin in response to LPS. Although LPS has been identified as a major regulator of hepcidin in other cell types, including alveolar macrophages and hepatocytes [7], we were unable to detect a response in our NHBE cells despite the addition of CD14 to augment the ability of the airway epithelial cells to respond to LPS [29].

We chose to study the regulation of hepcidin by IFN-γ in airway epithelial cells, because like hepcidin, IFN-γ provides an important link between immune response and iron homeostasis. IFN-γ acts as a principal affecter of cell mediated immunity by promoting the internalization of bacteria by macrophages and stimulating their elimination. IFN-γ decreases the expression of the transferrin receptor and ferritin [30,31] and thus, contributes to antimicrobial defense by limiting iron availability. IFN-γ classically signals through the JAK-STAT pathway [32], such that binding to the IFN-γ receptor phosphorylates JAK2. Phosphorylated JAK2 subsequently phosphorylates and dimerizes STAT1, which translocates to the nucleus. The hepcidin promoter has been reported to contain a putative binding site for STAT proteins [26]; therefore, we investigated IFN-γ regulated hepcidin expression via the JAK-STAT pathway through the activation of STAT1. We were able to demonstrate a marked and sustained phosphorylation of JAK2 as well as phosphorylation and nuclear translocation of STAT1 in the presence of IFN-γ. Further, blocking JAK2 phosphorylation with the chemical inhibitor, AG490, partially inhibited IFN-γ-induced hepcidin expression, suggesting that activation of STAT1 is required for full IFN-γ-regulated hepcidin gene expression in airway epithelial cells. This is consistent with induction of hepcidin via STAT1 activation that has been described in a murine macrophage cell line [33]. The lack of complete inhibition of IFN-γ-induced hepcidin expression in our cells also implies that additional signaling pathways are required [34]. This is reminiscent of the coordinated transcriptional activation of hepcidin in response to IL-6, which requires activation of both the STAT3 and BMP-response elements [35], and supports the idea that complex transcriptional regulation of hepcidin is required for precise coordination of iron availability in the face of inflammation [36].

IFN-γ is known to stimulate the production of pro-inflammatory cytokines, including IL-6 [22,23]. Hepcidin expression is strongly regulated by IL-6 in a number of cell types [26,37]. We were able to demonstrate that although IL-6 is capable of inducing IFN-γ in our cells, it does not contribute to IFN-γ-mediated hepcidin expression. Rather, IL-6-induced hepcidin expression occurred much earlier than that mediated by IFN-γ, suggesting that these two inflammatory cytokines may provide a concerted response to infection [35,38], and thus an additional means to fine tune the regulation of hepcidin.

While IFN-γ-responsive hepcidin expression was present in airway epithelial cells, we were unable to demonstrate a change in cell surface expression of Fpn or an accumulation of total intracellular iron in the presence of exogenous, biologically active hepcidin. The localized defense of the lungs requires a coordinated response of both airway epithelial cells and alveolar macrophages to effectively scavenge excess iron from the airway and sequester it from invading microbes. Given the role of the Th1 cytokine, IFN-γ in defense against intracellular pathogens by macrophages [7,39], we next investigated whether hepcidin produced by airway epithelial cells acts on alveolar macrophages to coordinate iron sequestration. Again, we were unable to demonstrate changes in total cellular iron accumulation in alveolar macrophages in the presence of exogenous hepcidin. The lack of internalization of cell surface Fpn in macrophages in response to hepcidin is consistent with previous investigations. RAW264.7 cells treated with IFN-γ together with *Salmonella typhimurium *demonstrated increased hepcidin gene expression but also increased Fpn gene expression and an overall decrease in the cytoplasmic iron content. Indeed, addition of exogenous transferrin-bound iron to these infected macrophages resulted in increased survival of the *S. typhimurium *[40]. Further, infected murine bone marrow macrophages treated with exogenous hepcidin demonstrated internalized cell surface Fpn, increased cellular iron retention, and enhanced growth of intracellular organisms [41]. Bone marrow-derived macrophages and RAW264.7 macrophages have been reported to increase hepcidin expression in response to IFN-γ, but only in synergy with the intracellular organisms *Mycobacterium avium *or *M. tuberculosis*. This increase in the hepcidin expression was localized to mycobacterium-containing phagosomes [10]. While iron transport was not measured in these cells, the authors demonstrate direct antimicrobial activity by lysis of the *mycobacterium *[10]. This suggests that cellular localization of hepcidin in response to invading pathogens, which is not well assessed by our methodology, is an important component of the immune response of resident cells of the airway.

Although the primary function of hepcidin is considered to be the regulation of iron homeostasis, hepcidin was initially identified by its close structural and functional resemblance to the defensin antimicrobial peptides [3,15]. Hepcidin shares the common amphipathic secondary structure and the net positive charge that are displayed by defensins and other antimicrobial peptides. This structure allows permeation of the membrane of invading microorganisms and results in a broad spectrum of antimicrobial activity [3,15]. Inducible antimicrobial peptides are an important component of the innate immune system. They are predominantly expressed at epithelial surfaces where interactions with the outside environment exist; this includes the airway [16]. Similar to defensins, hepcidin has been shown to provide direct killing of gram negative and gram positive bacteria as well as fungi [15]. Therefore, the antimicrobial function of the hepcidin peptide may provide a more direct mechanism for airway defense. Further investigation is warranted to determine if increasing the expression of hepcidin in the airway can provide direct antimicrobial effect *in vivo*.

## Conclusions

The airway is continually exposed to inhaled microbes and environmental particulates and therefore must be able to respond to local factors to impact iron metabolism and immune function. To achieve this, epithelial cells and macrophages of the lung have developed coordinated defense mechanisms that include mucociliary clearance, modulation of macrophage activation, and secretion of antimicrobial peptides [42,43]. Here we have demonstrated that airway epithelial cells express the iron regulatory peptide hepcidin in response to IFN-γ and IL-6. Hepcidin has been identified as a peptide that serves as both a regulator of iron homeostasis and an antimicrobial peptide. In our study, we could not demonstrate a direct effect of hepcidin on cellular iron regulation under the conditions studied. However, as a defensin-like molecule, hepcidin has a broad spectrum of antimicrobial activity in addition to its iron regulatory functions, which may provide an important anti-microbial defense in the airway. Further study may be warranted to determine the mechanism of hepcidin's direct anti-microbial effects and to determine if these effects are impacted by altering cellular iron localization within airway epithelial cells or macrophages.

## List of abreviations

NHBE: Normal human bronchial epithelial cells; ALI: air liquid interface; IFN-γ: interferon-γ; Fpn: ferroportin; JAK: Janus Kinase; STAT1: Signal tranducer and activator of transcription 1.

## Competing interests

The authors declare that they have no competing interests.

## Authors' contributions

MF and LM carried out experimental work and drafted the manuscript. AG participated in experimental design and participated in manuscript preparation. JT initiated the study, designed the experiments and participated in manuscript preparation. All authors read and approved the final version of the manuscript.
